# [Retracted] Everolimus inhibits the proliferation and migration of epidermal growth factor receptor-resistant lung cancer cells A549 via regulating the microRNA-4328/phosphatase and tensin homolog signaling pathway

**DOI:** 10.3892/ol.2025.15133

**Published:** 2025-06-10

**Authors:** Xudong Xiang, Li Zhuang, Huicheng Chen, Xiumei Yang, Heng Li, Gaofeng Li, Jing Yu

Oncol Lett 18: 5269-5276, 2019; DOI: 10.3892/ol.2019.10887

Following the publication of this paper, it was drawn to the Editor's attention by a concerned reader that certain of the cell migration assay data shown in Fig. 1D on p. 5271 had already appeared in an article written by different authors at different research institutes that had already been published in the journal *Thoracic Cancer*.

Owing to the fact that the contentious data in the above article had already been published prior to its submission to *Oncology Letters*, the Editor has decided that this paper should be retracted from the Journal. After having been in contact with the authors, they accepted the decision to retract the paper. The Editor apologizes to the readership for any inconvenience caused.

